# Developing an International Occupational Therapy Service: Perspectives and Implications

**DOI:** 10.3390/healthcare9111466

**Published:** 2021-10-29

**Authors:** Estíbaliz Jiménez-Arberas, Luis-Javier Márquez-Álvarez, Isabel Fernández-Méndez, María-Luisa Ruiz-Fernández

**Affiliations:** Degree in Occupational Therapy, Faculty Padre Ossó, University of Oviedo, 33008 Oviedo, Spain; estibaliz@facultadpadreosso.es (E.J.-A.); isabelf@facultadpadreosso.es (I.F.-M.); luisa@facultadpadreosso.es (M.-L.R.-F.)

**Keywords:** international cooperation, international agencies, occupational therapy, empowerment

## Abstract

Mali is one of the poorest countries in sub-Saharan Africa. Limited infrastructure renders access to health care difficult. There is a need to establish functional ways to improve Malian people’s health and treat disability. From this point of view, our project aims to implement a remote occupational therapy service for the beneficiaries of the Kalana clinic in Mali through international cooperation. Using a spiral iterative model, a proposal for a remote occupational therapy service was developed and refined for a multidisciplinary context. The International Classification of Functioning, Disability, and Health (ICF) was used as a means to work from a multidisciplinary approach to treat all needs. The results are exemplified with a case report and qualitative impressions of the services.

## 1. Introduction

Numerous organisations, foundations, and agencies have dedicated effort and attention to the developing world and development cooperation. One of these is Medicusmundi, a non-governmental organization (NGO) that works on issues relating to health and advocates for “health as a universal right”. Since it was founded in 1963, the NGO’s projects have adapted to the contextual conditions present in the communities where it works, helping to promote human rights and involving the community in all its activities.

Medicusmundi works in South America and Africa. Among its projects is a programme to improve the health of the inhabitants of Kalana, Mali [1]. World Health Organisation (WHO) reviews via the Health Resources Availability Mapping System to determine the availability of basic health services display an increase in health facilities from 2013 to 2016 but also reveal the difficulty of hiring staff, the need for ongoing training, the lack of equipment, and the inadequacy of financial resources [2]. Mali is 179th of 188 countries in the Human Development Index, according to WHO reports [3]. In terms of social indicators, it is one of the poorest countries in sub-Saharan Africa and has been classified as a “least developed country” by the Development Assistance Committee. Its main health indicators show a high prevalence of HIV (1.1%), one of the principal targets of health policy. According to the WHO data, 85.2% of the feminine population suffers from genital mutilation. Its causes are mostly cultural rules and patterns, and they are sustained by a religious context. The data also reveal an infant mortality rate of 10% and a neonatal mortality rate of 7.5%. Limited infrastructure renders access to health care difficult, and only 1 in 3 births are attended by health workers. Meanwhile, 13% of children under 5 years old are malnourished. Malnutrition is linked to 35% of cases of infant mortality, as well as to the emergence of disabilities and developmental problems, leading to participation difficulties [4].

The Kalana health centre is open every day, although most appointments are made on a single day of the week, coinciding with the local market day, while children attend the school located near the health centre. The centre provides malnutrition prevention services and treatment for health problems [1]. Given the scattered nature of the population, cases are generally monitored on a monthly or bimonthly basis. Access to the health centre is often difficult for families, as parents must travel with their child and leave the rest of their children in the care of the husband’s other wives.

The role of rehabilitation professionals in responding to conflicts and disasters is constantly evolving, and the development of our professions is intertwined with global events that have generated the need to provide care for overwhelming numbers of injured people [5]. In 2018, 14,000 individuals were assisted, including 230 children. The total population potentially requiring treatment is 36,081 people spread across 31 different villages [1]. Through international cooperation, the Bridging Horizons through Occupational Therapy project aims to implement a remote occupational therapy service for beneficiaries of the clinic and child nutrition centre in Kalana. Kalana is a mining and cultivating village; it belongs to the Guandianka—Yanfolila area of the Sikasso region. Its borders are with Guinea, closest, and Ivory Coast. Its climate is humid subtropical most of the year. Because of mining, Kalana has grown with different populations from all places in the country, now having mixed ethnicities and languages. There are two big mines being technically exploited, but hundreds of mines are manually exploited, which puts the lives and health of children and young and adult people, either women or men, at risk.

Occupational therapy has the merit of being extremely broad in scope and flexible in application across different populations, locations, and areas of occupational dysfunction. Occupation is an integral part of the human condition and an innate nature that we all share. The use of occupation transcends culture and is a determinant in health [6]. Professional evidence in occupational therapy has increasingly been characterised by a social, political, and cultural approach encouraging critical, reflective reasoning in the search for new paradigms [7]. However, in the context of international cooperation, few initiatives attempting to establish occupational therapy services have been documented [8,9].

People with disabilities are part of the most vulnerable population of Kalana. From a cultural perspective, according to our contact in the health centre, people there must be useful or productive, and disability is understood as a punishment. When a family go to work, people with disabilities are frequently left in a hole in the ground until night or the family returns. Rehabilitation centres, although helpful, are very few and too far away. In addition, it has been even more difficult to access these people since the recent armed conflict.

This project aims to implement a remote occupational therapy service for the beneficiaries of a Kalana clinic in Mali through international cooperation. Occupation-based modalities can span the whole continuum, from trauma to social reintegration and the eventual rebuilding of civil society, while creating a space where meaning and healing can be given tangible shape [10]. The project relies on the collaboration from Oviedo (Spain), with teachers from the Occupational Therapy degree programme and Occupational Therapy undergraduates, as well as working professionals in health care and other associated disciplines, working together in partnership with the health centre in Mali (via telematic assistance). All of them participate altruistically on a voluntary basis.

## 2. Materials and Methods

The project is based on an iterative model. Iterative design is a methodology based on a cyclical process of prototyping, testing, analysis, and amendment of a product or procedure. The results from each test are used to refine the process as much as possible, providing increasingly effective results.

### 2.1. Iterative Process

**Figure 1:**
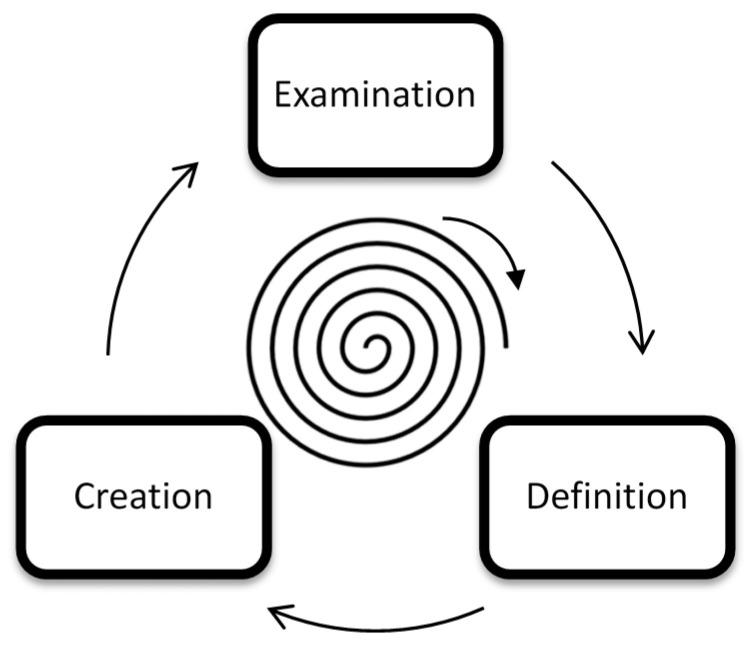
Basic outline of spiral iterative design based on Goodman [11].

Using an iterative design, this project aims to implement a remote occupational therapy service protocol that would be perfected over time through social engagement with Kalana (Figure 1) [11].

Although there is no evidence that this type of methodology has been used to design intervention protocols on prior occasions, both the method and the protocol share the idea of a progressive cycle, which refines itself through data-driven development [11]. In this project, the spiral iterative model is viewed as an alternative to the main quantitative and qualitative methodologies (such as phenomenological or narrative). From this point of view, it was thought that an ethnographic approach from the qualitative method was useful for the adaptation of the assessment tools, or even the research-action approach, which is very similar to a spiral design. However, the reciprocity of the information was not sufficiently continued, and the team could not get deep enough into the information about the clients of the health centre. In these terms, an iterative spiral model prioritised the product; with the long times between the answers from the health centre with the information, it was possible to refine the process and assessment methods to the greatest possible extent.

The underlying approach draws on Iwama’s idea that “understanding the cross-cultural utility of occupation, as occupational therapists have discoursed it, holds profound implications for the current practice, research and the future outlook for this profession” [12]. By engaging in north–south cooperation, we are able to obtain results with clinical relevance, and it is therefore a key priority to establish the method.

The aim is not to produce statistically significant results or explore a phenomenon in depth, but rather to attempt to adapt a service to a culturally different context by means of a series of relevant practices.

#### 2.1.1. Step 1. Examination

In this step, the issues present are examined, and their severity is ascertained in order to assess needs. In the project, the different social determinants of health were evaluated in the Kalana context in an attempt to establish working objectives for an occupational therapy service that would care for people, especially children with developmental problems, caused in many cases by endemic malnutrition, who are treated at the clinic in Kalana. Using this facility, we worked with health professionals to study the needs of each individual child. For a multidisciplinary approach, the International Classification of Functioning, Disability, and Health (ICF) was used as a means to talk among the team in Spain.

In reference to ICF as a framework to measure health and disability, the document holds that disability is not an attribute of the person but rather a set of conditions, many of which are created by the social context. [13] The ICF provides a systematic framework for organising client needs and capabilities. It allows one to prioritise objectives in terms of functioning and disability and, on the other hand, contextual factors [14,15,16,17].

The ICF includes all activities, tasks, and roles that fall within the scope of occupational therapists’ professional skills. The Royal College of Occupational Therapists views the ICF as a shift in approach from a simple medical diagnosis to a consideration of an individual’s context-based problems [18]. Uses of ICF as a framework reflect the concepts and definitions currently used in occupational therapy, understanding individuals as occupational beings. Moreover, many of our working models and theories are linked to sections of the ICF.

An initial screening ICF core-set was made for this purpose, through a Delphi method created through a panel of experts in health sciences and students. All the participants of the group participated in little discussions about the preliminary result, which was subsequently modified to match the perspectives of the whole group. Now, the core-set collects information on 36 possible questions which relate to different determinants, from which the patient can be referred to different professionals (Table 1).

In later design phases, this work provided a baseline for analysing progress and making changes to the intervention protocol, data collection process, and tests employed, following a SWOT approach (Strengths—Weaknesses—Opportunities—Threats), a ‘looking in’ and ‘looking out’ approach that covers the internal organizational factors of strengths and weaknesses and the external factors of opportunities and threats [19].

#### 2.1.2. Step 2. Definition

Step 2 involves identifying solutions. This was carried out at team meetings attended by working occupational therapists, medical doctors (specialists in surgery, sport medicine, and internal medicine) and occupational therapy teachers and students. Adopting a multidisciplinary approach, with the perspective of the different ICF core-set issues collected, the cases were examined by the group with a view to identifying potential courses of action, which would be subject to change and reflection in order to refine the process. In the data collection and data analysis sections, more information about this step is added.

#### 2.1.3. Step 3. Creation

Solutions are implemented. The mechanisms required to carry out tests and collect results were agreed with the health centre staff. As services from the health centre are dispensed on an irregular basis, patients’ attendance at therapy sessions and data collection took place discontinuously. Therefore, guidelines for family members were prioritised and certain tests were adapted to the cultural context under joint supervision from the health centre staff, fostering an atmosphere of continual improvement. For example, within the strategies to treat a burn, because there was a need to massage the zone daily, families were trained to handle it, or if a developmental scale was used in children, toys like blocks could be changed to everyday objects, like rocks, wood, and so on.

The main results indicators used were specified in each case report, with different qualitative and quantitative results; the related scales involved are included in the data analysis. In addition, the degree of cultural or contextual knowledge of the day-to-day situation in Kalana obtained by each of the group participants, the number of tools adapted to the context and culture, and the number of times that each child returned to the health centre for an appointment were added. The final indicator refers to family perceptions of the evolution of the case; the team proposed that, as if the child gradually improves, the family will continue to visit the health centre and receive a greater number of services, like educational ones.

As stated before, this phase does not mark the end of the cycle; the results produced can be used to analyse new approaches in the following testing phases. In terms of effectiveness, and with the aim of refining each process, results will be revised in a new examination step, with a SWOT approach of the process.

### 2.2. Data Collection

The creation of an intervention protocol for data collection was a priority for the working group. In order to foster inclusion among the different figures and professional profiles on the work team (from Spain: four occupational therapists, five occupational therapy students, two doctors and a nurse; from Mali: two nurses and assistants of the health centre) and establish effective communication with the health centre in Mali, the Standards of Practice for Occupational Therapy from the American Occupational Therapy Association (AOTA) were adopted [20].

**Figure 2:**
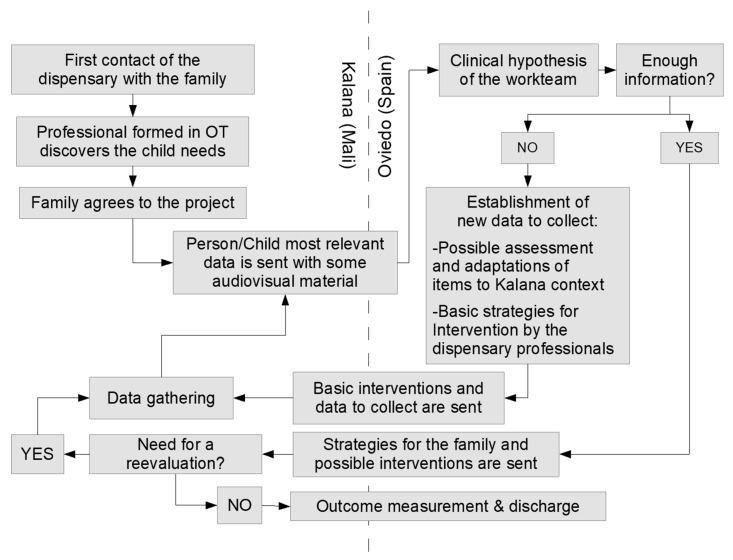
Data collection protocol.

The results of the methodological process described above are summarised in the protocol in Figure 2. It is important to note that the protocol has changed considerably since interventions began, and that part of it is still subject to change to allow for continuous improvement. These different protocols of remote care are the core of the international collaboration between Oviedo (Spain; Europe) and Kalana (Mali; Africa).

### 2.3. Data Analysis

**Figure 3:**
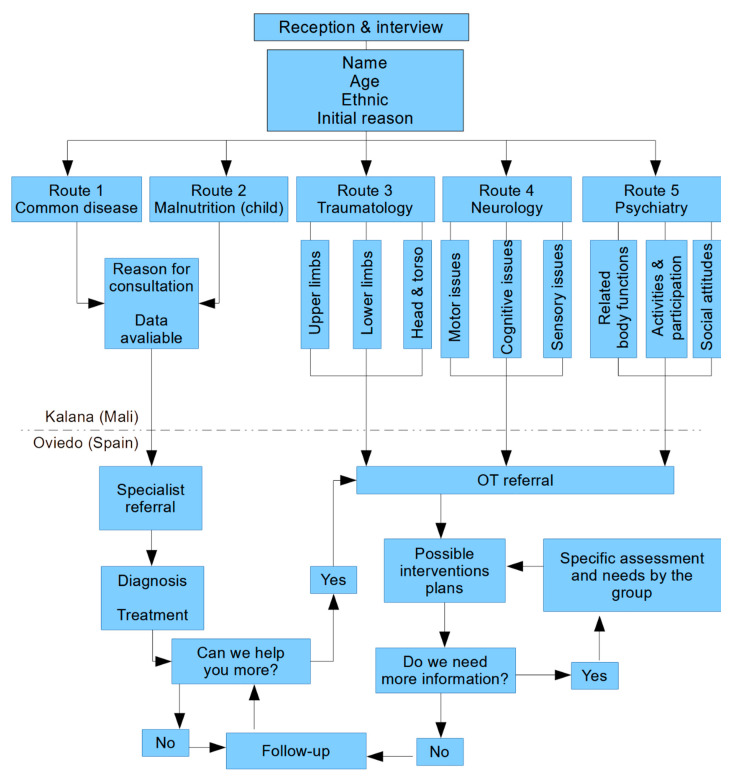
Data analysis protocol.

Data analysis was designed in a similar way to the data collection protocol. Starting with an initial interview and clinical data collection to analyse each case, different protocols were created and translated into French by a specialized interpreter to assure the correct analysis for every person. Figure 3 shows the protocol for data analysis.

As Figure 3 shows, data analysis was based on the iterative model in its composition, as it tries to develop and refine each of the procedures to collect data while also trying to make the data collection process easier to improve the quality of the occupational therapy procedure in terms of time. We collected and analysed data through five different “routes”. Routes were the name given to the possible protocols or guidelines which could enable us to establish a multidisciplinary approach for a client-centred practise. In these terms, choices 3 to 5 were specific to occupational therapy. Each participant of the project was classified in one of these routes to distribute the work among the team. A responsible group of volunteer students looked for the best evidence and translated it into ways to intervene in the Malian context, trying to offer an individualised plan of intervention. In addition, thanks to this, the students have clinical cases to work on, under the supervision of a practician.

Route 3 was created to collect data from bone, soft tissue, or joint damage. This route collected information in terms of (a) localization of damage, (b) restrictions of movement, and (c) integrity of tissues. From a biomechanical perspective, the analysis was performed through the collection of evidence (photos) and the localization, which allowed the team in Spain to classify them through the Vancouver Scar Scale [21] and Patient and Observer Scar Assessment Scale [22]. Because the personnel in Kalana did not know about how to use a goniometer technically, the results were based on evidence of evolution (as tissues recovered) and on a task-oriented approach, in terms of what he/she could not do before and can do now.

Route 4 was established to cover the neurological damage spectrum of possible case reports. It was divided into three possible issues: motor, cognitive, or sensory. (a) Motor data analysed possible plegias (thanks to illustrations) and muscular tone (Ashworth scale); cranial nerves, which were briefly examined for discarding any evident dysfunction; walking, which was assessed in terms of needed help, coordination, and strength (Daniels and Worthingham Scale) [23]; as well as pathological reflexes and hand function (Sollerman Hand Function Test) [24,25]. (b) For cognitive issues, a version of the Montreal Cognitive Assessment [26] was culturally adapted to gather information about short-term and long-term memory, language, spatial and temporal orientation, and attention. (c) For sensory issues, a brief questionnaire with qualitative data collection to cover all possible sensory modalities was used. For kids, we adapted the Haizea–Llevant scale to recover all the needs in terms of normal development [27].

Route 5, for mental health cases, was the last incorporation to the protocol, as it was more difficult to get a consensus. We incorporated a qualitative questionnaire about different ICF categories: (a) related body functions, like temperament and personality, impulsivity, sleep, attention, memory, psychomotor, emotions, perception, and so on; (b) activities and participation like problem-solving, making decisions, routines, stress management, care of own health, familiar and intimate relations, roles and occupations; and (c) environmental factors, with special relevance to social attitudes.

Data was analysed from an individual perspective to stablish more culturally adequate interventions, again following the SWOT approach. From the iterative model perspective, there is a need to constantly refine each part of the process, so the team must work with the evidence to analyse which results have a better fit to the user, and understand the feedback of the client and the family and how the process has been managed. Table 2 summarises these routes with the suggested main tools and the ICF issues related.

### 2.4. Ethical and Legal Aspects

The project obtained the approval of the Ethics Committee of the Principado de Asturias Public Health Services (ethics approval number code CEImPA 2020.091).

One of the main concerns in the project was ensuring anonymity when processing personal data and safeguarding the right to privacy and informed consent. The legislation applied in the project was Spanish Organic Law 3/2018 of 5 December on the Protection of Personal Data, the Guarantee of Digital Rights and European Regulation (EU) 2016/679 of the European Parliament, and the Council of 27 April 2016 on Data Protection.

For those individuals who were unable to read or write, a video declaration of their consent to participate in the project was recorded, while their rights were read to them in French. They were required to indicate their consent by saying “oui” at the end. Permission from both legal guardians (if the child had two) was required for each of the children who participated in the project and their decision-making capacity was protected by encouraging them to state whether or not they wanted to form part of the project if they were able to do so.

The sensitivity of the data was taken into consideration, and data were anonymised or pseudonymised in order to protect individuals’ privacy and preserve their rights. In addition, using the protocol described in the Results section below, the data minimisation principle was applied from the design stage: only the minimum data required to formulate a hypothesis and strategies for intervention was collected.

## 3. Results

### 3.1. Intervention in Clinical Cases

Intervention was carried out in a total of 15 clinical cases from the start of the project (May 2019). Most of the patients were children (*n* = 13) because the health centre in Kalana distributes nutritional supplements to prevent malnutrition in children. The characteristics of the centre make it easier to monitor the people attending it, most of them families with children. Each academic year allowed the distribution of the project periods (from September to July), and we tried to attend all the cases; there were up to five cases in study simultaneously through the course, with a maximum response time of 2 weeks with assessment or intervention lines. From May 2020 to February 2021, there was an impasse in the follow-up due to COVID-19, public health issues, and guerrilla and civil wars. This made it difficult to establish any line of intervention. As of October 2021, in the present period (since September 2021), we have added one more case.

During the initial appointment, health centre staff note the children’s developmental needs and problems. They then contact the group with the initial screening data.

In this context, the administration of assessment tests and teaching of specific technical approaches to intervention was hindered by distance; we attempted to overcome this challenge by sending all documents via Telegram, which offers a data protection policy that is acceptable to all members of the group.

The resources used for the project include only the human resources (the team itself and the families) and one computer, sent to the health centre for video-meetings. However, thanks to contact with different NGOs, it was possible to send to Mali a camera, Wi-Fi availability, cohesive bandages, and assistive devices for ADL, along with the costs of translation of documents and a brief stay in Oviedo for the head nurse of Mali, in order to establish the project and give her training in some occupational therapy techniques.

Another aspect relating to the clinical cases is cultural relevance. Drawing on each case, protocols and evaluation/intervention plans are adapted to reflect the cultural characteristics of Mali. For example, the tools used to satisfactorily administer the Haizea–Llevant scale [27] are adapted to incorporate items commonly used by children or available at the health centre.

The incompatibility of standardised scales and the individual assessment of each specific case made it difficult to adopt a common measure. For this aspect, it has been decided that the Goal Attainment Scaling (GAS) system will be used for the intervention plan [28]. This allows a transdisciplinary approach to be taken and objective changes to be identified.

Feedback became an invaluable tool for improving the project and refining the intervention protocols, as communication was very difficult with the health care centre (in terms of health crisis, guerrilla, etc.). To date, intervention continues in five different cases. Four cases have been discharged. Assessment results are pending in three cases, although no intervention has yet been carried out.

### 3.2. Case Report Example

AB is a 3-year-old-boy who came to the health centre because of different burns on his head and upper limbs. As the initial protocol of data collection, information was sent to the group, with photos of the child and the main problems. According to our data analysis, it was included in route 3. Our group concluded by the photos that almost 28% of the body had first- and second-grade burns caused by boiled water (head and left upper limb), which had subsequently scarred over. There was a persistent rigidness in the hand, with closed fist; in the face, his left eyelid could not be closed, and his mouth was not able to open, close, or move. He spoke by a little lateral gap between the lips and could not fed or maintain properly the food in his mouth.

The urgency of the case made the group give indications to the family for scar treatment while recovering more information about the adherence of the scars, functionality of the hand, and pain of the skin. According to the information returned, because of the eyelid damage, there was reduced visibility and an excessive dryness of the eye, which could lead to ulcers. We trained the personnel in Kalana in the Galveston technique, Kinesio Tape, and cohesive bandage through a specific protocol, and she gave the appropriate indications to the family about the use of both cohesive bandage and k-tape (the health centre could buy them to provide it to the family) to treat the adherence of the scars and eyelid, as well as the indications regarding cleaning and hydration of the scar to avoid limitations in joint range and mobility. Once a month, or once every two months, the family returned to the health centre so we could provide new indications about improving the mobility of the hand and the feeding.

After one year of intervention, the child can now feed properly, holding the food within the mouth with a good use of the orofacial musculature without losing it. He can close his eye voluntarily and is able to do grasp and precision grips to participate in his daily living activities with the rest of the family. With this, he can join his family life.

## 4. Discussion

This project describes an approach to international cooperation in which services are provided in areas with fewer occupational opportunities using widely accessible resources. Considering the social determinants of health and health inequities, Mali presents a unique context not only in terms of the structural determinants of health inequalities, and not only in terms of the socioeconomic and political context (it currently is in civil war), but also in terms of their own axes of inequalities, for example according to gender (female mutilation in 92% of cases) [29]. In addition to economic and social actors, material resources are minimal, as are health services, among which there are hardly any health professionals trained to serve citizens, including rehabilitation professionals. Hence, the need for this project arises, and given the current impossibility of carrying out international cooperation in situ, this project perspective on empowering clients and personnel in Mali was developed.

The testimonies of the families showed positive feedback up to now about the multicultural experiences. Both professionals’ and participants in Kalana’s views are very hopeful, which helps to maintain a positive relationship between them and the health professionals in Mali. As Cower-Ripley et al. state, the use of telerehabilitation reduces health care costs, improves treatment adherence, and enhances patient physical, cognitive, and mobility function, as well as patient satisfaction and health-related quality of life [30]. We firmly believe that our preliminary results showed congruence with this trend. Therefore, projects of this kind not only raise awareness of health professions, but also draw on an appropriate theoretical framework and scientific evidence in our field.

Guidelines have been identified for effective remote rehabilitation practices in international cooperation contexts, along with barriers and limiting factors. Among the most relevant are the stigmatisation of disability in the Malian context, as well as in other parts of Africa, and the excessive duration of interventions or indications and monitoring over time, as families visit the health centre once a month on average.

Regarding the methodology, there is no evidence that it has been applied to the creation of health or occupational therapy services on previous occasions, but the results are promising. By extrapolating this methodology to non-traditional contexts, we can identify new approaches to the creation of clinical intervention programmes. Measuring results in terms of these methodological aspects, with a greater roll-out of the protocols and implementation of an effective response time, could improve participation from different organisations in a broader context.

According to the World Federation of Occupational Therapy [31], if we include work on international cooperation in the “Disability, inclusion, and participation” category, it is largely carried out by universities. However, it is neither a priority area, nor does it have a large number of scientific publications. This article may serve as a model for establishing new avenues for collaboration between universities and occupational therapy associations and organisations seeking new intervention protocols in international development cooperation contexts to improve the accessibility and supply of existing facilities. Guajardo states that occupational therapy builds knowledge and expertise within its context as a social institution but never beyond it, so it must contribute to resolving social issues [32]. The inclusion of disabled people at the family, community, and societal level is linked to economic, cultural, and political factors [33], which should be explored in our field. This is the case of our project, which could provide new experiences to optimise existing resources and to incorporate the dynamic of review into individuals’ routines. The protocol created is gradually adapted to cultural factors and included within a routine, seeking to connect the occupational resources offered by the Kalana context. Despite the fact that the current global pandemic situation has meant that many rehabilitation services have had to adapt to a “virtual” intervention, the work we present has a methodological design to, among other objectives, recruit data and adequately carry out the measures of results derived from the intervention in a single context such as Mali and from the discipline of occupational therapy, which is aimed at the occupational performance so important to all the inhabitants of the planet, but which—in a context such as the one that is exposed, that is not “independent and useful“—runs the risk of being abandoned.

Despite the limitations, its application has successfully improved the occupational participation of patients at the Kalana health centre, bringing the project closer to achieving its principal objective. According to Hasselkus, the embeddedness of occupation in human lifespan development may be the most powerful dimension of the relationship between occupation and well-being [34]. In this cultural context, disability has particularly severe consequences as individuals’ participation in their nearest physical surroundings is limited to their capacity for immediate productivity; a child who is unable to participate in daily chores is relegated and marginalised from the community. It is therefore crucial to improve the availability of resources. Resources may exist but are not considered as available to some people for personal and/or context-related reasons [35].

International action allows pathways towards occupation and social participation to be forged, not only through in-person intervention but also through the creation and coordination of new resources [36], learning itself [37], and the internationalisation of professional development [38]. Our work with the community in Kalana is based on observation and intervention with people, as well as on African cultural understandings of the community, family, and communal life. The wellbeing generated may be enhanced through intervention with individuals, but it also requires consideration of their families and wider contexts [39]. This is the ultimate aim of the guidelines for families. The health centre staff will not be direct practitioners; instead, they will act as a tool for empowering patients attending the clinic and encouraging them to make their own changes to their environment.

### 4.1. Future Lines of Research

This project is currently being extended to meet two main needs: on the one hand, the creation of an application to improve the transmission of information from north to south and enhance the confidentiality of the data for patients, and on the other, the supply of sufficient material to the health centre in Kalana in order to establish more effective intervention programmes.

Due to the short time the project has been on, and because of the difficult time that has been this last year and a half (with COVID-19 and the war situation in Mali), there is a plan for starting the assessment of the whole project from September 2021. Consensus chose GAS [28], with long-term and short-term goals, as the best means to do so. Short-term goals are stablished in semesters, and long-term goals use the whole academic year (as the organization of the group operates around it).

These measures are intended to improve the intervention, making it quicker and enabling closer monitoring and better coverage. The inclusion of additional professional profiles in the project reflects the same aim, as expanding the areas of expertise covered could improve the intervention and allow us to work more holistically with the community.

From a subjective perspective, the disability concept needs to change in Kalana. The health centre demands as a line of investigation research about the cultural understanding of disability. In our society, people with disabilities have been protected by families, associations, and groups, governments, or laws. It seems very relevant to determine these factors in looking for a change of mind in the Mali context that could preserve occupational justice.

### 4.2. Methodological Considerations and Limitations

Our purpose as professionals was to create an effective remote intervention protocol adapted to the different contexts present in Kalana and to attempt to exploit as many resources as possible depending on their effectiveness. Due to the characteristics of the methodology, results could not be measured quantitatively, and the project was supplemented with a qualitative section exploring the perception of these results among all the components of the project (families, personnel, and volunteers).

However, the additional difficulty of the intervention time has direct repercussions on the number of possible results obtained. It is not currently feasible to record a result greater than that obtained from the intervention itself, with a very small sample size and highly sporadic interventions. Therefore, our aim is to continue to evaluate the project for at least three more years in order to produce statistically significant results.

## 5. Conclusions

Preliminary results suggest that this protocol design could improve the quality of life of Malian people with disabilities. The use of ICF is an effective resource to promote the multidisciplinary communication among teams and allow the project to be relocated in other countries in need of health services.

## Figures and Tables

**Table 1:** Questions for the initial screening, related to the created core-set.

Component	Questions
Body functions	Does he/she need third persons’ help or an assistive device to move?
	Does he/she move in an independent way over short distances?
	Does he/she move in an independent way over long distances?
	Can the person lift, move, or manipulate objects of different weights with his/her upper limbs (according to his/her age and physical skills)?
	How is his/her posture in standing and sitting positions?
	Has he/she difficulties in planning his/her activities?
	Can he/she decide by him/herself?
	Has he/she inadequate behaviours or emotional responses related to his/her chronological age?
	Does he/she clearly manifest an inadequate physical development related to his/her chronological age?
	Does he/she have peer relations?
Body structures	Does any absence of one or more limbs exist? (Specify which(s) one(s).)
	Does any presence of burns and/or scars exist? (Specify places.)
	Does any malformation in one or more parts of the body exist?
	Does any other structural alteration in any one or more parts of the body exist?
Activities and participation	Is he/she capable of learning and retaining that knowledge?
	Is he/she capable of applying that knowledge in activities of daily living (ADL)?
	If he/she has any difficulty in any activity, is he/she capable of doing it by imitating?
	Which is his/her communication modality?
	Is he/she able to complete a simple task independently? (For example, choose the clothes to dress, take them, prepare them, and get dressed.)
	Is he/she able to complete an age-related routine independently?
	Does he/she utilise his/her hands to manipulate little objects?
	Does he/she involve both hands in his/her ADL?
	Is he/she able to wash and groom him/herself with autonomy?
	Does he/she maintain proper hygiene in his/her body without anyone else remembering it?
	Is he/she continent (urinary and faecal)?
	Is he/she autonomous to eat and drink?
	Has he/she any access to formal education? If yes, until when?
	Does he/she participate in community activities?
Environmental and personal factors	Has he/she access to necessary food and drink to survive?
	Has he/she access to necessary objects to do his/her ADL (including productivity)?
	Has he/she access to communication devices, like mobile phones (including assistive technology)?
	Has he/she access to necessary materials for his/her education?
	Does he/she own a home?
	Distance from his/her home to the clinical resource.
	Frequency assisting at the clinical resource.
	Possibility in accessing clinical assistance.

**Table 2:** Summarised assessments and outcomes measures in protocol routes.

Route No.	ICF Related Issue	Tool/Assessment	Outcomes
3	s810, b810, b820, b760	Observation (photos):	Evidence of evolution
		(a) localization of damage	
		(b) restrictions of movement	Task-oriented skills (what he/she could not do and can do now)
		(c) integrity of tissue	
4	b760, b730, b735, b740, d410–d429, d450–d469	Plegias schemes	Pre-post results (quantitative)
		Ashworth scale	Quality of gait and walk
		Cranial nerves	
		Daniels and Worthingham Scale	
		Sollerman Hand Function Test	
	b114, b140, b144, b167, d175, d177	Montreal Cognitive Assessment (culturally adapted)	Pre-post results
	b210, b230, b235, b250, b255, b265, b280	Sensory screening	Qualitative data related to participation in ADL
		(for kids only) Haizea–Llevant scale (culturally adaptated)	Cues acquisition
5	b126, b130, b134, b140, b144, b147, b152, b156	ICF brief questionnaire (additionally included: roles and most meaningful occupations)	Qualitative data changes related to participation in ADL
	d175, d177, d230, d240, d570, d760, d770		
	e410–e465		
